# Implementation of Falls Management Exercise (FaME) falls prevention programme among community-dwelling older adults. A scoping review

**DOI:** 10.1186/s12889-026-27772-9

**Published:** 2026-06-13

**Authors:** Sally Hoodless, Jodi Ventre, Sarah K. Smith, Helen Hawley-Hague

**Affiliations:** 1 Division of Nursing, Midwifery & Social Work, School of Health Sciences, Faculty of Biology, Medicine, and Health, The University of Manchester, Jean McFarlane. Oxford Road, Manchester, M13 9PY UK; 2 Manchester Academic Health Science Centre (MAHSC), Manchester, M13 9NQ UK; 3 Applied Research Collaboration - Greater Manchester (NIHR ARC-GM), National Institute for Health and Care Research, The University of Manchester, Manchester, M13 9NQ UK; 4 School of Health and Social Care, The University of Bradford, Bradford, UK

**Keywords:** Strength, Balance, Exercise, Falls prevention, Implementation, Older adults, Technology, Teleconferencing

## Abstract

**Background:**

A common cause of injury, disability, mortality, and health-related costs, falls affect a third of adults aged 65 years and older. Falls Management Exercise (FaME) is an evidence-based, progressive falls prevention programme, shown to significantly reduce falls and concerns about falls among community-dwelling older adults. Despite the proven benefits of FaME, there are inconsistencies in implementation fidelity and programme delivery. Variances in attendance, adherence and dose, required to ensure efficacy, can negatively impact real-world effectiveness of FaME.

This scoping review aims to address the paucity of evidence synthesis on FaME implementation. Identifying key characteristics relating to intervention delivery, implementation and behaviour change strategies, participant uptake, adherence, meeting of effective dose, attrition, and fidelity.

**Methods:**

Adopting an approved methodology for conducting scoping reviews, seven electronic databases were searched using the Population, Context and Concept inclusion criteria. These relate to; older adults (≥60 years), ‘community-dwelling’ and FaME. From an initial 10,888 papers, 26 research papers (13 studies), were included in the full text review, of which ten papers (six studies) used technology in delivering FaME.

**Results:**

Key themes: challenges in meeting recruitment target and uptake of FaME, particularly underserved ethnic and socioeconomic populations; significantly fewer older men participating in FaME when compared to older women; issues of poor adherence and meeting of effective dose, especially with home-based exercise components; high rates of attrition and the value of Instructor qualities in delivering FaME. Studies which included technology-assisted implementation reported increased participation by older men, with programmes performing better in terms of adherence, attrition and exercise dose.

**Conclusions:**

With digital health technologies showing promising effects on persistent challenges to adherence, dose, and attrition, technology-assisted implementation of FaME has potential to effectively support a wider uptake of FaME among community-dwelling older adults who decline or withdraw participation. Further research is needed to increase our understanding of under-served community-dwelling older adults experiences, and perceptions about FaME and falls prevention. We need to further explore the acceptability of technology-assisted implementation of FaME, and feasibility for supporting a wider roll-out of FaME across the United Kingdom.

**Clinical trial registration:**

Not applicable.

## Background

Falls are a substantive and prevalent cause of injury, morbidity and mortality among older adults [1]. Accounting for the second greatest cause of unintentional deaths globally (estimated at 684,000 fatal falls per year), adults over 60 years of age experience the most fatalities [2]. Non-fatal falls resulting in injury, for example a hip fracture, can precipitate residual short or long-term disability, negatively impacting on activities of daily living and increasing the likelihood of long-term residential care [3, 4]. With fall-related hospital admissions escalating exponentially with age [5], the socio-economic healthcare impact of falls in the United Kingdom (U.K.) is significant, costing more than £2.3 billion each year [6]. Whilst not all falls cause major injury, being unable to get up after a fall is identified as a predictor of significant fall related injuries from future falls, suggesting a poorer prognosis, with a greater risk of hospitalisation, long-term care admission and mortality [7, 8]. Psychological effects associated with concerns about falling can perpetuate a negative trend in fall risk and well-being [9, 10], leading to longstanding problems with loss of confidence and independence [11]. With a predicted rise in older people over 60 years estimated to double to 2.1 billion by 2050, falls are a global health concern [1]. Importantly, falls are not inevitable and can be prevented. Progressive, structured strength, balance and functional programmes, delivered by appropriately trained professionals are recommended for all older adults, and people 50 years and over who are at higher risk of falls [12].

Falls Management Exercise (FaME) is an evidence-based, progressive, strength and balance falls prevention intervention proven to reduce rate and risk of falls [13]. Endorsed by NICE [12], promoted by The Department of Health and Social Care as an example of good practice [14], and strongly recommended by the World Health Organization (WHO) [1]. Delivered by specialist instructors, trained through Later Life Training (a not for profit organisation providing evidence-based exercise training for health professionals working with older people) [15], FaME is proven to be effective in reducing the risk of falls, fall rate and concerns about falling, improving balance, and increasing habitual activity [13, 15]. As a cost-effective intervention, with a return of £2.89 to £50.59 for every £1 invested [16], FaME can benefit a wide demographic of community-dwelling older people at risk of falls. Originally trialled as a secondary preventative programme for frequent fallers, it is also effective as a primary prevention intervention [13, 17–19]. Referred through both primary and secondary care services, or through self-referral, participants are invited to attend the six month programme (24-weeks). Consisting of weekly face-to-face group classes, delivered by specialist trained Postural Stability Instructors (PSI), with skills to work specifically with older people at risk of falling, the programme is supplemented with additional home-based strength and balance exercises to meet the 50 h of physical activity recommended for falls prevention [15, 16, 21]. Building on Otago Exercise Programme (OEP), these exercises progress the balance and strength components beyond the original OEP intensity, including upper body strength activities [21, 22]. Incorporating the seven evidence-based key components of dynamic endurance, balance and targeted resistance training, functional floor activities, exercises for flexibility, adapted Tai Chi and retraining or maintaining the ability to get up off the floor to prevent detrimental long lays following a fall (backward chaining), FaME builds on these activities over three progressive phases; (1) Induction or Skilling Up Stage, (2) Training Gain Stage and (3) Maintaining the Gains Stage. Tailoring progression over this extended period accommodates individual ability, giving the necessary time to achieve training effects [13, 16, 21]. Despite the proven benefits of FaME, there are inconsistencies in its implementation, delivery, availability, attendance, and difficulties in maintaining efficacy, motivation, and retention, with limited data on the quality of the programmes [23].

The aim of this scoping review is to address the scarcity of evidence synthesis on the implementation of FaME for community-dwelling older adults. It seeks to explore, and map available evidence in the research literature [24], identifying participant characteristics, facilitators and barriers to uptake, implementation strategies and fidelity (the extent to which FaME is delivered as intended by the evidence base), challenges in programme delivery and gaps in existing knowledge. The results of this review may inform future research, with the potential to support a wider roll-out and uptake of FaME across the United Kingdom.

## Methods

### Search strategy

A comprehensive scoping literature review was conducted, directed by approved methodology [24–26]. Seven electronic databases (CINAHL Plus, MEDLINE, EMBASE, ProQuest, Scopus, PsycInfo and Pubmed.gov) were searched for peer-reviewed literature in the English language up to and including articles published until May 2025. Additional publications were identified from manual searches, reference lists of eligible papers and citation searching.

**Fig. 1:**
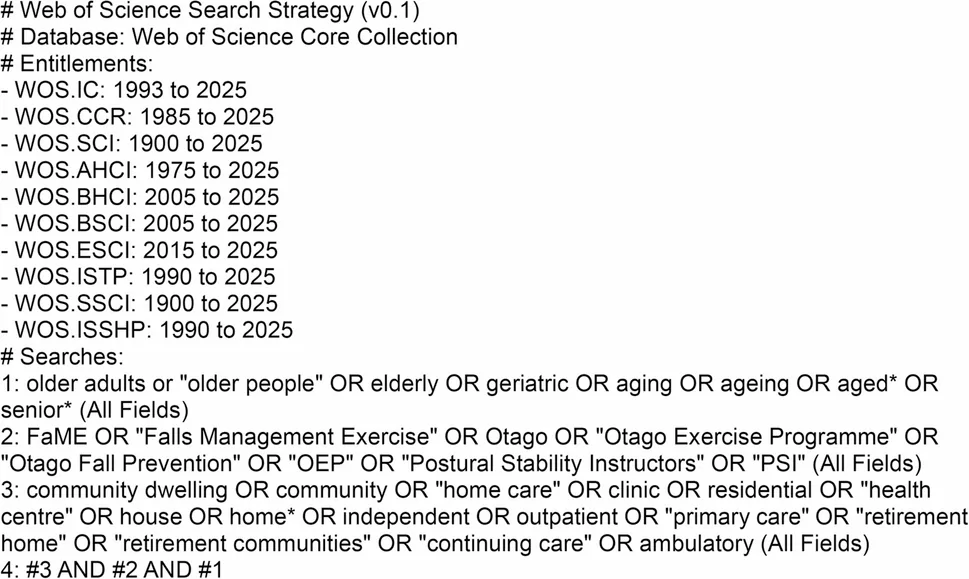
Web of Science Search Strategy (v 0.1)

Maximising the search strategy, theoretical frameworks for conducting scoping reviews [24–26] were used with guidance from experienced librarians and specialists in falls and aging research. Using Boolean operators, search terms were applied using the Population, Context and Concept inclusion criteria framework [26]. These related to the population of older adults (≥ 60 years) and Postural Stability Instructors, in the context of being community-dwelling, with the concept of the Falls Management Exercise (FaME) programme. An example of the full search strategy and terminologies applied for the Web of Science are presented in Fig. 1.

Studies which included secondary or residential care (FaME is designed for community-dwelling older adults) or assessed FaME only as part of a wider cost or outcome analysis of falls prevention interventions were deemed ineligible. Given the limited number of available studies, no date restrictions were applied. Published conference abstracts, research protocols or Editorials/letters were not included in the review but sought as necessary for supplementary information. Additional detail about the search strategy is included in the Open Science Framework (OSF) scoping review registration [27].

### Research literature search results

Three independent reviewers (SH, JV, SO’C) performed the blinded screening process, using reference management software, EndNote 20 and Rayyan research collaboration platform. Intervention validity was clarified by an expert in falls prevention (HH-H). All studies not meeting the predetermined eligibility criteria were excluded. The final search returned 10,888 potentially eligible records. After screening and removal of duplicates, 26 eligible publications (13 studies/trials) were retrieved for full text data extraction. The majority of the papers (23) were from the United Kingdom, two papers from Sweden and a single study from Hong Kong. Articles were published between 2005 and 2025 inclusive. Research design included 13 randomised control trials, four feasibility and usability studies, three implementation studies, three evaluation studies, and three studies using a mixed methodological approach; an exploratory design, a cohort study with intervention and comparison groups and a patient preference comparison study. Sample size ranged from six to 1,417 participants, with ages from 59 to 101 years. The combined mean age of community-dwelling older adult participants was 75.65 years, congruent with the inclusion criteria. The papers were reviewed in accordance with the phases of the PRISMA 2020 flow diagram for updated systematic reviews which included searches of databases and registers only (see Additional file 1. Figure 2. PRISMA_flow_diagram_v1.0).

## Results

### Data extraction, evaluation and synthesis

The lead researcher (SH) used a pre-defined piloted extraction form for the data charting process [24], under the guidance of the wider team. The principal data extracted included; publication identifiers, study design, population characteristics, concept (intervention/comparator), context and mode of delivery, implementation strategies, behavioural change theories/approaches (including those embedded in FaME), adherence, barriers and attrition rates and associated variables. These extracted data are documented in greater detail in Table 1a and1b [see Additional file 2]. The charted data was synthesised using a textual narrative summary approach [28, 29]. Key findings were organised into themes which were refined into commentaries and titled; recruitment strategies and challenges, population sociodemographic characteristics (including underserved groups, health and socioeconomic status), the heterogeneity of FaME programmes, non-technology and technology-assisted implementation and fidelity, effective adherence and attendance, dose, attrition and exercise Instructor properties and experience.

The demographic data is summarised in the additional Table 2 (a and b) [see Additional file 2].

### Recruitment strategies and challenges

Strategies for identifying, approaching and recruiting older adult participants across the studies were varied. Identification, referral, participant approach and invite through the National Health Service (NHS) was employed by the majority of the studies. Patients were identified through medical records and invited to participate by General Practitioners and Primary care staff, integrated community falls rehabilitation services and physiotherapists working with older adults [20, 30–32, 36, 39, 40, 42, 47, 48, 50, 51]. Other methods included advertising or approaching patients in hospital clinics and departments [13, 30]. Non NHS recruitment strategies included advertisements at public community events, in other public and healthcare settings [19], through third sector voluntary organisations and using local and national public communication media to good effect [13, 30]. Recruitment through older adults community centres and assisted living facilities was adopted by Yeung et al. [19], Wytske and Stanmore [33], Stanmore et al. [45], and Mansson et al. [47, 51].

Less direct strategies utilised were the identification of people who had participated in previous related studies [38, 41], and the use of routinely collected secondary data from existing FaME programmes [22, 35]. Postural Stability Instructors (PSI’s) and Health Professionals were identified and approached through local falls services and Later Life Training [15, 39, 42]. This was through convenience or purposive sampling [23, 44, 49], and by using email, phone or face to face snowball sampling [23].

Several of the interventions had difficulty with, or failed to recruit to the target participant sample size, resulting in an extension of the recruitment period, recruitment sites and an increase in the number of people approached and invited to participate [31]. Recruitment rates ranged from < 10% of eligible people approached [20] to 78% [30]. Of the other studies which reported recruitment details, successful recruitment was below 70% of eligible people [19, 23, 30–36]. Where identified, reasons for declining participation or poor take-up were given as; too busy or competing priorities, caring responsibilities, not wanting to undertake trial activity or intervention, fear of technology, fatalistic view of falls, feeling too young to need a falls prevention programme or self-perceived low risk of falls (even if fallen previously), already attending group rehabilitation, or an unwillingness to complete home exercises [13, 19, 31, 36–38]. Underestimation of the time needed to recruit and organisational factors, such as room availability for baseline patient assessments at recruiting sites may have exacerbated the length of time it took to recruit, prolonging the start of the programme [31]. Allowing longer recruitment periods could be used to alleviate this problem [31]. The use of supplemental screening procedures can be employed to negate assessment of potential participants who do not meet the eligible criteria [31].

### Population and sociodemographic characteristics

#### Gender

All studies recording participant gender reported less men than women taking up the Falls Management Exercise (FaME) programme. The total mean of female and male gender across all of the studies was 69.96% and 30.03% respectively. The proportion of women varied from 42.8% [39, 40] to 100% of the recruited participants [13, 41, 42]. Whilst included in the total mean, it should be noted that the RCT and secondary data analysis conducted by Skelton et al. [13, 41] purposively sampled community-dwelling older women only. Two papers showed a better representation of male inclusivity ranging from 42% − 58% [38, 39]. The higher prevalence of female Postural Stability Instructors (PSI) as research contributors was also reflected in the studies by de Jong, et al. [38] at 100%, Hawley-Hague et al. [43] at 87% and Ventre et al. [44]. Whilst greater participation by older men has been reported with technology-assisted FaME programmes [30, 33, 36, 39, 40, 51], there is a lack of detail and evidence in the studies about the reasons for this incongruity.

#### Socioeconomic status

Studies that report on the socioeconomic status of the participants reported fewer participant groups being amongst the 20% most deprived areas in the U.K [23, 34, 35, 45]. Whilst this may not have been a contributing factor for inclusion or uptake of participants from deprived areas, the majority of studies did not indicate charging a fee for the classes or intervention [13, 19, 20, 23, 30–40, 42, 45–48, 50, 51] and free transport or reimbursement was also provided [30, 38, 42, 46]. When participants paid for classes, the cost was considered acceptable at £1.50 to £3 per session [49].

#### Ethnic diversity

Of the papers that documented ethnicity, the predominant groups were participants who identified as White, White British or White Irish or with English indicated as a first language, ranging from 87.7% to 100% inclusivity. This was also reflected by the PSI participant group in the FaME delivery study by Hawley-Hague et al. [43] with 87% of total participants identified as White British. There is sparse information in the papers for this disparity in participant ethnic diversity in FaME programmes. Few studies specified being able to speak, comprehend or follow instructions in English as part of the inclusion criteria [30, 33, 38, 45, 46].

### Comorbidities and perception of health

The incidence of comorbidities ranged from one condition to 13 or ‘multiple’ and the taking of three, to more than four medications in studies which included this data [20, 23, 30, 35, 36, 38–40, 46, 51]. When older adult participant groups were recruited from mixed sites, it was identified that self-reported health conditions and levels of physical activity differed between the recruitment sites [51].

### Falls risk, history of falls or reduction in balance

Fall history of participants was not consistently documented. In those which did, a history of falls, including having a fall within the past 12 months, affected the majority [36, 39, 40, 47]. Comparatively, the cohort who participated in the Exergames falls prevention intervention had a significantly lower prevalence of previous falls, multiple falls, and single falls than the control group [45]. In the study by Lafond et al. (2019) [48], the baseline data for both the FaME and OEP groups gave a falls risk median (Interquartile range) of 1.0 using the Falls Risk Assessment Tool (FRAT; range 0–5), indicating a lower risk of falls. The majority of studies used functional assessments at baseline, as part of the pre-assessment, and as outcome measures to assess balance and falls risk, functional reach, confidence in balance, concerns of falling, physical activity, health related quality of life and avoidance of activity. Tools used include, Timed up and go (TUG), Functional Reach, Falls Risk Assessment Tool (FRAT), ConfBal scale, Falls Efficacy Scale International (FES-I), SF-12, 30 s chair stand, 6 min walking test (6MWT), Berg Balance Scale-Short Form (BBS-SF), PHONE-FITT, Time to rise from floor, Tinetti balance and Black Fracture Risk Score [13, 17, 20, 23, 30–32, 36, 37, 39, 45–47, 50, 51].

### Implementation and fidelity of FaME

In the primary research studies, FaME was trialled either as a single intervention, alongside a comparator of OEP, as standard care/usual activities, or both. Most studies documented the inclusion of many of the key components of FaME in the implementation and delivery [21, 22]. However, across the studies, not all of the components were routinely delivered, as guided by the recently updated FaME implementation toolkit [15, 52]. Key components that were delivered included an initial participant health screening, pre-exercise assessment, (including goal setting and motivation to exercise), functional assessment, and evidence-based, individually tailored exercise programmes, necessary for ensuring participant safety and fidelity of the intervention [22]. Programme content that were omitted or less likely to be included were identified as; backward chaining, adapted Tai Chi cool down, PSI’s not asking participants about falls in the previous week, limited follow-up on non-attendance and not explaining the functional purposes or benefits of exercises [34, 42, 43]. When explored, reasons for the FaME deliverables being omitted were given as; functional floor work (including backward chaining), not normally offered or introduced as part of the rehabilitation programme, which may be in part due to the frailty of the patient and the relatively short treatment period [36, 39, 43], as well as lack of confidence (PSI’s and older adults) in teaching or progressing activities in groups of variable abilities; [30, 34, 43, 48], fall recovery information being provided in an education session [35], participant fear and tiredness, and time constraints [43]. Pre-exercise assessments were self-reported to be carried out by most PSI’s to tailor delivery or provide outcome measures [43], reflected in the study by James et al. [35]. Although identified by Stevens et al. [31] that assessments were not always completed using a standardised risk categorisation, other studies which explicitly documented the use of assessments used screening tools and assessments usually administered by the exercise instructors as part of the standard FaME programme [30, 46]. A number of papers gave reference to tailoring exercises as part of continuous safety assessments, for participants who were particularly deconditioned (e.g. using chair-based lower intensity options), subjectively determined by the trained exercise practitioner [35, 49], or influenced by participant self-perceived ability [34]. The challenges of standardisation and fidelity were expressed in a number of studies [30, 31, 34, 43, 46, 48, 49]. In particular, this was challenging when trying to meet the needs of all participants with different capabilities, varied functional abilities and rates of progression [31, 43] leading to some more advanced participants feeling that they weren’t being physically challenged enough [38, 46]. This was echoed by the programme Instructors, as was the need for an additional exercise professional to support participants with multiple comorbidities and those less physically able with functional floor work, especially in larger classes of mixed capabilities [30, 34, 43, 49].

### Community PSI led group classes and home-based exercises

Eight studies (15 papers), delivered standard, non-technology assisted face-to-face FaME group classes, led by PSI’s, with the recommended additional unsupervised home-based exercises (as previously described). The home exercise instructions were provided in a paper booklet format [13, 19, 20, 23, 31, 32, 34, 35, 37, 42, 43, 47–50]. Programme duration ranged from 24 weeks [49] to 36 weeks [13, 19], with the majority of studies delivering over 24 weeks [20, 23, 31, 32, 34, 48–50], consistent with current evidence-based guidance provided in the updated FaME Implementation Toolkit for Commissioners [15, 52]. Research to understand FaME delivery in the U.K. saw participants joining classes from six weeks to an indefinite length of time [43]. Across the papers, a varied range of venues were used for class delivery. These include; community venues, GP Practices, health and social care units, hospital, sheltered housing/assisted living and other locales such as day and leisure centres [13, 19, 20, 31–33, 35, 37, 42, 43, 46, 48–50].

### Technology-assisted implementation of FaME

The use of technology to assist in the implementation of FaME was described in seven studies (ten papers). The majority of studies used technology as a hybrid approach to complement in-person delivery of FaME and support home-based FaME and OEP components [30, 33, 36, 38, 39, 45, 46]. Non-hybrid feasibility studies used technology to remotely deliver evidence-based exercise programmes to participants in their own home following a short period of standard care and/or physical pre-assessment [40, 47, 51].

A wide spectrum of available technology was employed. Audio clips (using a CD player, MP3, or DVD machine) verbalising the home-based exercises were delivered as part of an adapted standard in-person PSI led FaME programme for visually impaired older adults [29, 45].

Smartphone apps supporting standard service were co-developed and tested by Hawley-Hague et al. [36, 39]. The ‘Motivate Me’ (clinician app) and ‘My Activity Programme’ (patient app) was used to support home-based FaME and OEP exercises alongside in-person, evidenced-based rehabilitation therapy (group and individual). Personalised goal setting was transferred between the clinician and patient apps allowing individualised two-way feedback on exercises completed at home, reviewing of goals, messaging and prompts to exercise.

Meekes and Stanmore [33] and Stanmore et al. [45] used strength and balance exercises constructed from OEP and FaME in the Exergame (videogame) programme, implemented using the MIRA system and a Microsoft Kinect platform with in-person physiotherapy support for participants at a sheltered housing location.

Delivered remotely to participants in the home, a digital programme, using FaME inspired web-based or mobile application, was accessed through a tablet, phone or computer by participants in the self-managed preference trial by Mansson et al. [47, 51]. Consisting of short video clips of exercises imaging older adults completing the exercises, with verbal instructions and virtual physiotherapy feedback, level of progression was self-determined by the participant [47, 51].

A single study used teleconferencing to deliver real-time remote health professional led standard individual rehabilitation home exercises, and a group-based strength and balance programme. Incorporating OEP and FaME components through a smartphone system whilst patients remained in their own homes [40].

Technology-assisted interventions all had a shorter study period than those not using technology, except for Hawley-Hague et al. [36] that delivered the programme up to 6 months.

Barriers to technology-assisted implementation were around usability, confidence with technology, feasibility, internet provision, financial cost of connectivity and availability of patient owned technology (e.g. smartphones), particularly amongst those living in the more deprived areas [39, 40].

Gender differences around technology identified women participants having greater access to smartphones (65%) than men (47%) but with female participants reporting less access to computers than males 49% and 84% respectively [47].

The Technology Acceptance Model (TAM), a validated tool for predicting acceptability of a technology intervention was used as a questionnaire by Meekes and Stanmore [33] and to help inform the interview schedule in the study by Hawley-Hague et al. [40]. TAM uses the constructs of perceived usefulness (the degree to which a technology will improve performance) and perceived ease of use (the effort needed to use the technology) to anticipate if a technology will be rejected or accepted [53].

### Adherence, attendance, and dose

Measuring adherence to the intervention was included in most papers. However, the methods used to collect this data varied between studies. Those using technology-assisted delivery of the intervention used group exercise registers or number of sessions attended [30, 46, 51], participant self-reporting of home exercises [45–47, 51], and the Exercise Adherence Rating Scale (EARS) and self-reporting of exercises through an App, with the addition of issue log and field note data [36, 39]. Most prevalent in the papers using non-technology assisted delivery of the FaME was collecting attendance data. This was through daily diaries [13, 17, 20, 31, 32, 37, 50] and class attendance registers or recording of attendance rates [19, 20, 23, 32, 34, 35, 37, 42, 50]. Further detail about the adherence measures, including exercise intensity in Moderate-to-Vigorous Physical Activity (MVPA) minutes and adherence presented as a percentage of completed sessions is documented in Additional File 2.

Common throughout the studies, challenges of poor adherence to the FaME programme are identified, particularly in completing the home-based exercises and in meeting the recommended minimum effective exercise dose of ≥ 150 min per week [54]. Of the papers which analysed the exercise minutes completed, all reported below the minimum effective dose rates.

Reasons given for non-compliance or not meeting effective dose when undertaking the home exercises include difficulty adhering to home-based exercises, that they were not seen to be as challenging and were less enjoyable than the group sessions. That participants felt that they were less important, they missed peer support, social contact and camaraderie, lacked self-discipline to motivate and challenge themselves, cognitive memory issues and being unsure about the relevance of the exercises [42, 48, 49]. Lack of time and competing priorities were seen as additional barriers [42], although encouraging participants to incorporate the home based exercises into their daily activities and routines could improve participant motivation [42, 49].

When compared with usual care or control groups, technology assisted implementation was reported as achieving greater dose and adherence than the non-technology assisted comparator. Adherence varied from non-technology-assisted 40% [20, 23] to technology-assisted 87.8% and 100% for Exergame implementation, which although was technology-assisted, the delivery also included the presence of a physiotherapist during the activity and was not delivered fully remotely [33, 45]. Dominant facilitators supporting adherence, ascertained in both standard and technology-assisted studies were social factors, including family, spouse and group peer support, participant self-efficacy, text message prompts, goal setting and feedback, with tailoring and progression of exercises [13, 19, 32, 33, 36, 38–40, 42, 43, 45, 46, 49, 51]. Some studies also made specific reference to instructor characteristics (experience and knowledge) [13, 42, 43, 48, 49].

### Attrition

Where reported, there was a high incidence of participant attrition and withdrawal, both prior to and after randomisation. In studies that did not use technological devices in their implementation, the rates of attrition were reported as between 21% [19] of the total participants to 60% in the ‘real-world’ effectiveness and implementation study by Orton et al. [23]. Most reported causes of attrition by participants were related to cognitive decline and physical ill-health problems. Costs associated with transport provision, travel difficulties, public transport safety fears and cost, along with the proximity of the venue were factors identified by PSI’s delivering FaME [43]. Missing participant data, lost to follow-up, and reasons unknown for attrition and withdrawal were identified in some of the studies [20, 23, 32, 34, 35, 37]. Comparatively, the majority of technology-assisted implementation of FaME resulted in less attrition. In the study by Mansson et al. [47, 51], the self-managed FaME inspired digital programme, consisting of short exercise video clips with verbal instructions reported a 17% withdrawal rate compared to a 37% loss of participants using the paper booklet delivered intervention. The exception was the study by Adams et al. [30] which reported a 12.12% attrition in the intervention group, with no loss from the control. The reasons for this are not explored in the study. The comparison of gender, attendance and attrition for non-technology and technology-assisted Falls Management Exercise (FaME) are documented in, additional Table 3 (a and b) [see Additional file 2].

### Implementation and Behavioural Change Techniques (BCTs)

The studies in this review demonstrate inconsistencies and a lack of clarity about implementation strategies and Behavioural Change Techniques (BCTs) used in the intervention. Whilst many of the studies mention BCTs, few were mapped to Michie et al. BCT Taxonomy (v1) [55] with the exception being the papers published by Hawley-Hague et al. [36, 39, 40]. However, whilst not specifically identified as BCT’s in the majority of the papers, a number of elements grouped in the Taxonomy (v1) [55] were included. These groups were identified as; goal setting and planning, feedback and monitoring (including self-monitoring), social support, shaping knowledge (e.g. skilling-up phase), natural consequences (e.g. relating functional exercises to daily activities), comparison of behaviour (e.g. through demonstration), association (e.g. use of prompts for home-based exercises), repetition and substitution (particularly with home-based exercises and training gains phase), comparison of outcomes (e.g. benefits of exercise outweigh the costs), reward and threat (e.g. self or social reward), scheduled consequences (e.g. when completing full sequence of backward chaining), and self-belief (e.g. self-efficacy).

Descriptions of implementation theories that referred to the Expert Recommendations for Implementing Change (ERIC) [56] were similarly absent from many of the studies or otherwise not described or reported, common to clinical and falls prevention research [57, 58]. Those described include; multi-agency and patient collaborations using advisory boards and workgroups, preparing participants to be active in their care (e.g. ask questions about the relevance of the exercises to their daily lives), recruit, designate and train for leadership (e.g. in the specialist training for PSIs), use of media (for participant recruitment), distribute educational materials (e.g. in the form of home exercise instructions for participants and the updated FaME Implementation Toolkit for stakeholders [15, 52]), tailoring strategies, academic partnering, promote adaptability whilst maintaining fidelity and quality monitoring [13, 20, 23, 30–32, 34, 36–38, 40, 42, 43, 45, 46, 48, 51].

### Exercise Instructor characteristics and experience

Common themes around the importance and value of instructor characteristics were identified and seen as motivators for long-term adherence. The importance of bonding and building relationships with individual participants was recognised by some instructors [43] and participants [49]. Positive PSI traits include familiarity with the participants, being friendly, inclusive, supportive, enthusiastic and encouraging. Skills such as rapport building, sharing personal experiences and empathising, making exercise delivery fun and effective planning and organisation contributed to participants continued attendance [42, 43, 49]. This is reflected in the opinions of PSI’s who felt that personality was important for long-term adherence to exercise by community-dwelling older adults [43]. The comprehensive, specialist training that PSI’s complete prior to delivering FaME is necessary for successful programme delivery [21]. This includes the skills and ability to individually tailor and modify exercises for participants with varied degrees of ability and comorbidities [44]. Maintaining standards through Continuous Professional Development (CPD) and evidence-based training with appropriate activities helped PSI’s deliver the programme with confidence and fidelity. Instructor experience, imparting knowledge, being able to answer questions and provide positive reinforcement and support were values also viewed by both PSI’s and participants as important factors in the delivery of the programme and as motivators for participants to complete home exercises and maintain class attendance [43, 48, 49]. Conversely however, it was recognised by some PSI’s that a strong relationship with the participants may have the negative effect on fidelity, masking poor practice [43].

## Discussion

With the paucity of synthesised evidence on real-world implementation of FaME, this scoping review is the first to explore and map a body of literature on FaME research studies aimed at identifying key characteristics relating to recruitment challenges, effective delivery and implementation, participant characteristics and uptake, adherence, meeting of effective dose, and attrition. Many of the studies document challenges in meeting recruitment targets of eligible participants, and of those recruited, many participants were women, with men and minority ethnic older adults accounting for a smaller sample of participants. Measurement of fidelity to the programme demonstrated that implementation did not always adhere to the evidence-base. Behaviour change techniques were often a key component missing from intervention delivery, with only a small number of included studies mapping behaviour change techniques to the behaviour change taxonomy [36, 39, 40].

Research and real-world studies show that the FaME programme, when delivered with fidelity can significantly reduce falls and concerns about falling, and facilitate an increase in physical activity among community-dwelling older adults [13, 19, 20, 23, 32]. However, the findings of this scoping review suggests that it is not sufficient to rely solely on the efficacy paradigm of an intervention to determine intervention reach or representativeness of setting and participant communities in the real-world environment [59, 60]. Exploring why community-dwelling older adults decline participation in group exercise programmes can help to widen reach and improve equability of falls prevention interventions [37].

### Recruitment strategies and challenges

Recruitment challenges emerged across all included studies and are comparable with other reviews. In the augmented Cochrane Report (2012), by Nyman and Victor [61], on average, one in every five community-dwelling older people invited to take part in falls prevention RCTs were not recruited due to non-response, refusal or exclusion (not meeting trial criteria). Where reported, reasons for not meeting the recruitment target sample reflect many of the known challenges and barriers of recruitment to community falls prevention and strength and balance training interventions [62, 63]. Introducing telephone pre-screening of potential participants, may reduce the number of inappropriate referrals, mitigating previous time spent reviewing ineligible participants [31]. In providing detailed data about recruitment strategies, evidence-based recruitment planning for future studies and interventions could be facilitated [62, 65].

### Population and sociodemographic characteristics

#### Gender

The significant disparity between the higher proportion of women than men participating in FaME programmes corresponds to previously documented trends in falls prevention studies and health promotion activities [54, 66–68]. There is a lack of detail about the reasons for this incongruity in the papers reviewed.

Previous research which has sought to understand the underrepresentation of men in falls prevention, self-management health or exercise programmes suggest reasons for this disparity may be linked to the belief that women are considered to be in greater need of falls prevention, the influence of societal gender perspectives, group classes being viewed as more feminine, with socialisation appealing less to men than women [62, 69, 70]. Addressing these differences indicates the potential for dedicated recruitment strategies, including use of language, introductory sessions, and male fitness trainers, with an emphasis on leadership and structure and other delivery style approaches, for example, online, to appeal to older men [69–72].

#### Socioeconomic status

Participants stratified as amongst low to middle deprivation (10%−40%) are also not widely represented in the FaME studies identified. Financial hardship may prevent inclusion in fall prevention programmes for older adults who are unable to manage the associated costs, even with free provision of the programme [76]. Mitigating the risk of under-recruitment in studies from these diverse communities, and amongst low to middle deprivation may be through integrating existing relationships within communities [65].

From the demographic information reported across the studies, less people lived alone than with a spouse. Studies suggest that people who are not married are less likely to participate in physical activity than their married counterparts, and place more emphasis on their exercise preferences, including aspects on advice giving, monitoring and evaluation, group social activities, cost, location, and time of exercise than married counterparts [77, 78]. This may impact on older community-dwelling adults taking up and adhering to falls prevention classes such as FAME, with set days and times of attendance, travel requirements and unfamiliar venues.

#### Cultural sensitivity

Across all the studies, participants who were non-white, non-English or for whom English was not their native language (written or spoken) were in the minority. Ethnic inequalities across healthcare and barriers to access are widely reported. Influencing factors which may prevent programmes being culturally inclusive include diverse languages and availability of translation services, cultural understanding of exercise, cultural norms, direct and indirect discrimination, and geographical and socioeconomic inequalities [73–75].

### Co-morbidities and perception of health

Many of the studies excluded community-dwelling older adults due to pre-existing co-morbidities which are common health contraindications to physical activity [79]. Impacting on risk of falls in the general population, there is a 35% increase for each additional co-morbidity or incapacity, for women aged > 60 years, rising steeply from 8% for women < 60 years of age [80]. Additional risk factors for falls specific to men are identified as depression, older age, and poor balance [81]. As such, it would be pertinent for these older adults to be referred to FaME as and when health conditions and safety allow.

### Implementation of FaME

Adherence, attendance, and meeting of effective dose was suboptimal in many of the studies. Recognised as a challenge in falls prevention interventions, Nyman and Victor [61] concluded that at 12 months, only half of community-dwelling older adults will be adhering to falls prevention research programmes. With an absence of reported BCTs and implementation strategies, the fidelity, reproducibility, sharing of knowledge, and ability of an intervention to promote successful implementation of FaME and change behaviour long-term may be limited. Additional challenges are presented by real-world implementation studies, having less available resources, and with greater reliance on routine service procedures and outcome data [34]. In the studies which did not meet the recommended exercise guidelines, this was predominantly due to poor adherence of home-based exercise prescription. The challenges in meeting effective dose for falls prevention interventions, particularly for home-based exercise recommendations is documented, hampered by poor motivation, lack of confidence and distractions [82]. The importance of social influences, peer support, social responsibility to other participants, and the role and experience of the instructor are all recognised to have a positive impact on participant attitudes, attendance, and overall adherence [83]. Fidelity to the group intervention can be optimised by introducing in-service quality assurance systems, and observations of classes by experienced instructors [31, 34].

Existing attendance and adherence literature supports many of the common facilitators reported in the FaME studies. High self-efficacy is seen as an independent predictor of the intent to continue participation in long-term exercise maintenance, with lower risk of lapse and relapse [87–89]. Recognising the physical and psychological benefits gained from completing the exercises can also facilitate continued adherence [42, 49].

### Attrition

A common challenge throughout the studies, reasons for attrition predominantly included cognitive and physical health issues, caring responsibilities, lack of motivation and self-discipline and venue facilities [49]. This is comparable to the wider literature which also recognises transport issues, distance to venue, and a lack of understanding of relevance of the fall’s prevention programme and importance of completing the exercises [64].

### Technology-assisted implementation of FaME

FaME programmes which used technology-assisted implementation were shown to perform better in terms of adherence and exercise dose. Using technology to assist and enhance the implementation of FaME has shown promising favourable associations with adherence, dose, and attrition, alongside positive feasibility, usability, and acceptability. Systematic reviews have perceived eHealth and mHealth to be acceptable, enjoyable and engaging for older adults, positively impacting on adherence [84–86].

Whilst the benefits of technology-assisted implementation is recognised, it is noted that characteristics of the study participants (where described), reported better health, with higher levels of pre-intervention exercise for those in the digital or technology-assisted intervention arms [51]. This may suggest potential for selection bias, with greater access to technology [51], pre-intervention attitude being more positive and with people choosing to participate in the technology-assisted intervention, causing an overestimation of motivation [33, 51].

Potential benefits of using technology in the implementation of FaME components include reduced need to travel, cost effectiveness, use of goal setting, increased communication, support and confidence (self-efficacy), feedback, self-management, and flexibility [30, 33, 36, 39, 40, 45]. Providing additional support through the digital programmes may create better opportunities for acceptance and adherence long-term [51].

### Summary of evidence

#### Limitations and strengths

Multiple search platforms and databases were used to identify the studies, however, only those in the English language were selected. Not restricting the year of publication helped to mitigate omission of papers, in addition to several publications being identified either manually or through the citation chain. There is heterogeneity in the quality, methodology, and reporting of the primary studies in terms of implementation methodology, sample sizes, duration, data collection and inconsistencies of the reporting of outcome measures. There were also differences in FaME delivery. All programmes included key components of FaME, but not all delivered FaME with fidelity [13]. Whilst this limits synthesis of the findings and ability to form definitive conclusions, or inform practice, it is reflective of how FaME is often delivered in rehabilitation and the community. Variations and inadequacies in methodological rigor or reporting is suggested as being a specific issue of implementation studies [90]. The studies identified took place in two European countries, with predominantly white, moderately affluent English speaking, older women and a single study in Hong Kong. This limits understanding about the applicability of the FaME programme, particularly for underserved communities.

#### Considerations for future research

Of the studies included in this review, participant recruitment challenges were present and often below target for fall preventative interventions. More women took part across the intervention than men, and an even smaller number of older adults from minority ethnic backgrounds accounted for numbers in recruitment and participation. Future research should work with a diverse range of participants at risk of falling and eligible for the falls management programme to be able to understand further the barriers to participation, adherence and attrition for these specific currently underserved groups. Upon the completion of this work, evidence-based recruitment strategies may then be recommended that directly increase participation in the programme and enhance equity of access to underserved populations. Improved uptake of these interventions following effective transfer from falls rehabilitation to specialised strength and balance programmes are important. Continued support in facilitating self-management falls prevention for the older adult, through evidenced based strength and balance programmes such as FaME, can mitigate the revolving door of needing to re-access therapy services following a decline in strength and balance or further falls for community-dwelling older adults, following completion of their rehabilitation programme [91].

## Conclusions

Underrepresentation of certain populations in the uptake of FaME may impede a wider roll-out, limiting equitability, generalisability and reach. A lack of information about recruitment difficulties, and ethnic, socioeconomic and gender disparities across the studies limits the opportunity to understand the causes of underrepresentation. Adherence and dose, particularly with home-based exercises was suboptimal, with high levels of programme attrition in most of the studies.

Further research is needed to have a better understanding as to why community-dwelling older adults decline participation in group exercise interventions [37], and to address the paucity of evidence exploring the complex factors that facilitate or challenge uptake and adherence in FaME.

When comparing non-technology assisted with technology-assisted FaME implementation, it is reported that a greater proportion of older men participate, with less attrition across all participants when technology is used. However, persistent challenges of poor uptake, adherence and compliance remain, and the heterogeneity of interventions, populations, length of programmes and fidelity impedes synthesis of the data, limiting understanding and application. With the expansion of Information and Communication Technology (ICT), there is potential for technology to augment with in person delivery, helping to address these challenges, effectively supporting a wider roll-out of FaME [83, 85]. With fewer research studies using technology to assist delivery of FaME, it is necessary to explore the views of community-dwelling older adults, Health Care Professionals and Postural Stability Instructors on the acceptability and feasibility of technology-assisted implementation of Falls Management Exercise (FaME). This will enable data to be collected for future research studying the potential to deliver FAME with greater fidelity, to improve uptake, dose, attendance and adherence of the Falls Management Exercise (FaME) programme across the U.K.

## Data Availability

Data is included within the manuscript or additional files.

## Abbreviations

FaME: Falls Management Exercise
NICE: National Institute for Health and Care Excellence
WHO: World Health Organisation
PSI: Postural Stability Instructor
LLT: Later Life Training
OEP: Otago Exercise Programme
IMD: Index of Multiple Deprivation
TAM: Technology Acceptance Model
BCT: Behaviour Change Techniques
ERIC: Expert Recommendations for Implementing Change
CPD: Continuing Professional Development
ICT: Information and Communications Technology
